# Effect of ultrasound treatment on quality parameters and health promoting activity of fish protein hydrolysates extracted from side streams of Atlantic mackerel (*Scomber scombrus*)

**DOI:** 10.3389/fnut.2024.1446485

**Published:** 2024-09-04

**Authors:** Janna Cropotova, Kristine Kvangarsnes, Turid Rustad, Janne Stangeland, Gabriella Roda, Melissa Fanzaga, Martina Bartolomei, Carmen Lammi

**Affiliations:** ^1^Department of Biological Sciences Ålesund, Norwegian University of Science and Technology, Ålesund, Norway; ^2^Department of Biotechnology and Food Science, Norwegian University of Science and Technology, Trondheim, Norway; ^3^Møreforsking AS, Ålesund, Norway; ^4^Department of Pharmaceutical Sciences, Università degli Studi di Milano, Milano, Italy

**Keywords:** Atlantic mackerel, ultrasound treatment, FPH, functional properties, quality parameters

## Abstract

Fish protein hydrolysates (FPH) obtained by enzymatic hydrolysis allows for smart valorization of fish side streams. However, further treatments are normally needed to enhance bioactive and functional properties of the obtained FPH. At present, the commonly used methods to improve functional properties of FPH include chemical and enzymatic modification. Chemical treatments often cause environmental problems, while the enzymatic modification method requires the use of quite expensive enzymes. In recent years, emerging technologies such as ultrasound treatment (US-treatment) have shown great potential in protein modification with high efficiency and safety, low energy consumption, and low nutritional destructiveness. In this study, high-power ultrasound treatments were applied to fish protein hydrolysates (FPH) extracted from Atlantic mackerel (*Scomber scombrus*) side streams to improve their quality parameters. The effect of three different treatments of 300 W, 450 W and 600 W at the operating frequency of 20 kHz for 10 min on the physicochemical, structural, and functional characteristics of FPH, were examined. The results have shown that with an increase in ultrasound power, the protein solubility of FPH increased linearly, and the changes were significant for all US-treated samples compared to control (untreated) samples. US-treatment significantly increased the degree of hydrolysis of FPH samples treated with 450 W and 600 W compared to control samples. The carbonyl content of FPH increased (significantly for 450 W and 600 W), while thiol groups decreased (significantly for 300 W and 450 W). This indicated that some US-treatments induced oxidation of FPH, however the values of the protein oxidation were low. Amino acid composition of FPH revealed that US-treatment increased the proportion of essential amino acids in the sample treated with 300 W and 450 W, but the increase was not significant. After the US-treatment, all FPH samples became lighter and less yellowish and reddish, which suggest potentially higher attractiveness to consumers. In addition, the *in vitro* antioxidant activity was assessed using the DPPH, FRAP, and ABTS assays and the cell-free dipeptidyl peptidase IV (DPP-IV) inhibitory activity was also measured. Moreover, these biological activities were measured at cellular level utilizing human intestinal Caco-2 cells. Specifically, the FPH capacity to lower H_2_O_2_-induced reactive oxygen species (ROS) and lipid peroxidation levels was used to measure its antioxidant activity. The findings suggest that *Scomber scombrus* hydrolysates could find use as ingredients for promoting health.

## 1 Introduction

By 2050, the world population is expected to grow by about one-third, raising the global need for alternative sustainable and renewable sources of high-quality protein (1). Fish contains high-quality protein that comprises all the essential amino acids required for adequate human diet, making it a complete protein source. However, considerable amounts of this valuable resource are lost or wasted during harvest, transportation, processing, and distribution before reaching the consumer table (2, 3). Underutilized fish parts, including head, fins, scales, bones, viscera, trimmings, skin, and other fish discards represent a valuable source of high quality protein compounds including all essential amino acids, small bioactive peptides and hydrolysates. The utilization of fish side streams as a source of protein compounds has recently become increasingly popular for various industrial applications (2, 4).

Fish protein ingredients are mainly applied in the food industry due to their high nutritional value and techno-functional parameters. These properties determine how proteins behave under various food processing conditions, such as heating, freezing, and mixing, which ultimately impact the quality, texture, and stability of food products. Fish protein hydrolysates (FPH) have been shown to possess good solubility, emulsifying, foaming and gel-forming properties (5, 6). Moreover, FPH show promising health-promoting properties, including antioxidant, ACE and DPP-IV inhibitory activities (7). There is currently a large interest from the food industry towards effective techniques or methods capable to modify the spatial structure of protein molecules to improve their techno-functional properties and stability during processing (8). The conventional methods used for protein modification comprise chemical, physical and enzymatic modification (6). In recent years, advanced non-thermal technologies such as ultrasound (US), pulsed electric field (PEF), and high-pressure processing (HPP) have shown great potential in protein modification not only when applied as pre-treatments before extraction, but also as post-treatments for quality improvement. As an alternative physical method of modification, they have many application advantages such as high efficiency, safety, non-invasiveness, low energy consumption, and low nutritional destructiveness (8, 9).

In recent years, there has been a growing interest in the application of ultrasound on proteins and peptides, specifically focusing on their techno-functional properties (8). The main highlights of this study compared with other studies on the use of US-treatment are related to the application of ultrasound post-treatment for modification of the physicochemical, structural, and functional properties of FPH extracted from Atlantic mackerel side streams. There are no similar studies published so far on the effect of ultrasound post-treatments on quality parameters of FPH. It is well known that ultrasound treatment may induce a number of sonomechanical and sonochemical effects on a protein/peptide molecule. The sonochemical alterations are caused by the modification of side groups of amino acids or/and breakage of peptide chains. The sonomechanical changes in a protein/peptide molecule are due to intensive molecular agitation, which may result in a temporary or permanent modification of the 3D structure of the folded protein (10). Considering the fact that both techno-functional and bioactive properties of peptides depend on their structure, the application of ultrasound treatment might affect their quality characteristics (10).

The main techno-functional parameters of FPH that may be affected by ultrasound treatments, are gelling, emulsifying, water holding capacity, degree of hydrolysis (DH) and protein solubility. FPH can act as emulsifiers, helping to stabilize emulsions and prevent the separation of oil and water-based ingredients in a food matrix (11). This property is crucial in the production of food products like dressings, sauces, and mayonnaise, where a stable emulsion is desired (11). Another important techno-functional property of FPH is their water holding capacity. Water holding capacity refers to the ability of protein ingredients to absorb and retain water molecules. This property is particularly significant in food processing and formulation, as it directly influences the moisture content, juiciness, tenderness, and stability of food products (5). FPH with high water holding capacity are desirable, as they contribute to the desired texture and juiciness in meat products, baked goods, dairy products, and many other food items. In muscle foods such as meat and fish products, for instance, the water holding capacity of proteins plays a crucial role in determining the tenderness and succulence (12). The degree of hydrolysis and protein solubility influence the overall quality, taste/bitterness, texture, and consumer acceptance of the final product (11). Degree of hydrolysis refers to the extent to which a protein molecule has been broken down into smaller peptides or amino acids through hydrolytic reactions (5, 13). A higher degree of hydrolysis generally leads to improved protein digestibility and bioavailability, which is significant for individuals with specific dietary needs or health conditions, such as infants, elderly, or patients with gastrointestinal disorders (7). Protein solubility, on the other hand, refers to the ability of a protein ingredient to dissolve in a given volume of water. This parameter of FPH contributes to the desirable sensory qualities of food products, such as smoothness, creaminess, and mouthfeel, as well as bioavailability and digestibility of peptides in a food matrix (5). Peptides that are insoluble in a FPH mixture can easily precipitate and result in grainy or gritty textures that are generally not preferred by consumers. Protein solubility affects the bioavailability and digestibility of proteins in food (7). For instance, soluble proteins are more readily digested and absorbed by the body, providing essential amino acids required for growth, repair, and maintenance of body tissues. The present study will investigate the influence of ultrasound post-treatment on techno-functional parameters of FPH extracted from Atlantic mackerel (*Scomber scombrus*) side streams. FPHs are gaining attention for their health-promoting properties, primarily due to their high content of bioactive compounds, mainly bioactive peptides. These hydrolysates can enhance immune function by modulating immune response (14). Results reported in the literature indicates that fish derived peptides can exhibit anti-inflammatory effects by inhibiting pro-inflammatory cytokines, which plays a crucial role in managing chronic diseases such as arthritis and cardiovascular disorders (15). Additionally, fish peptides have been associated with cardiovascular health by promoting lipid metabolism, reducing blood pressure, and improving endothelial function (16). Moreover, these bioactive peptides facilitate muscle recovery post-exercise by providing essential amino acids that aid in protein synthesis and reduce muscle soreness (17). Their antioxidant properties help counteract oxidative stress, contributing to cell protection and overall longevity. Hence, the incorporation of fish-derived hydrolysates and peptides into diets looks promising for enhancing overall health and preventing various chronic conditions. As a second objective, this study therefore aimed at evaluating possible biological activities of FPH and the influence of the different treatments. To achieve these objectives, a comprehensive assessment of the potential antioxidant and anti-DPP-IV properties of the FPH was performed. The direct antioxidant activity was examined applying *in vitro* antioxidant assay, including 2,2-diphenyl-1-picrylhydrazyl (DPPH), the ferric reducing antioxidant power (FRAP) and 2,2′-Azino-bis (3-ethylbenzothiazoline-6-sulfonic acid) diammonium salt (ABTS). Then, the efficacy of the FPH antioxidant activity to reduce intracellular ROS and lipid peroxidation was evaluated in Caco-2 cells, where the oxidative stress was induced by H_2_O_2_. In addition, the FPH capability to decrease the activity of DPP-IV was biochemically valuated.

## 2 Materials and methods

### 2.1 Preparation of fish raw material

Atlantic mackerel (*Scomber scombrus*) was delivered whole from a local fish processing facility (Fosnavåg, Norway) in insulated containers filled with ice in September 2022. The fish were immediately gutted and minced using a mincer with 4.5 mm hole size (Hobart A 200N), divided into batches of 1 kg, and immediately frozen and stored at −80°C until enzymatic hydrolysis.

### 2.2 Enzymatic hydrolysis

The enzymatic hydrolysis of the mackerel mince was performed in a 4 L closed glass bioreactor placed in a water bath at 52°C. Warm (50 ± 2°C) distilled water was added to the fish mince in a 1:1 ratio. The mixture was stirred at 150 rpm with an overhead stirrer. When the temperature of the mixture was 50°C, Alcalase^®^ enzyme (Sigma-Aldrich, Germany) was added at levels of 0.1% (w/w) of the raw material weight. After 60 min of hydrolysis, bones were removed by filtering the hydrolysate through a sieve before the enzymes were inactivated by heating up to 90°C for 10 min in a microwave oven. The mixture was cooled down to 30°C before being transferred to 1 L centrifugation bottles and then centrifuged at 4,100 g at 4°C for 30 min. The liquid fractions (lipids and water-soluble proteins) were separated from the insoluble fraction (sludge) in the following way. The liquid fraction was placed in a separatory funnel and allowed to settle, and then separated into oil and water-soluble proteins. The water-soluble protein phase representing fish protein hydrolysate (FPH) was collected and dried in the laboratory vacuum freeze-dryer (Labconco Freezone Console 12 L Freeze Dry System).

### 2.3 Ultrasound treatment

FPH samples were subjected to ultrasound treatment at 300 W, 450 W and 600 W with a 20 kHz probe (Sonics & Materials Inc., Danbury, CT, United States, model: VCX 1500). The probe has a vibrating titanium tip of 1.2 cm which was immersed in the FPH solution followed by its irradiation with an ultrasonic wave directly from the horn tip. The samples were previously dissolved in distilled water in a ratio of 10 g per 400 mL. Then, the ultrasonic probe was immersed 2 cm below the surface of the hydrolysate solution, and the sonication was performed with a pulse duration of 5 s on and 5 s off. FPH samples were treated for 5 min with the intervals of 5 s passive (rest) and active (treatment) phase each, and the temperature was monitored during the treatment. At the end of the sonication treatment, the temperature of all FPHs was in the range of 43 ± 2°C.

### 2.4 Proximate analysis of FPH

Total nitrogen was determined in FPH prior to US-treatment by using the Kjeldahl method, quantity of protein was calculated as 6.25xN (18). Content of water was determined gravimetrically after drying at 105°C for 24 h. Ash content was determined by incineration to constant weight at 550°C (19). Lipid content was determined mathematically due to very low amount of fat in FPH through deduction of total protein, ash and water content from 100.

### 2.5 Molecular weight distribution analysis

Freeze-dried FPH was diluted with Milli-Q (MQ) water to a concentration of 10 mg/mL. Then, 100 μL of the diluted FPH solution was further diluted with 900 μL of 10% acetonitrile in MQ water in an HPLC vial. Analysis was performed on an AQUITY UPLC H-Class PLUS System (Waters Corporation, Milford, MA, United States) with an AQUITY BEH125 SEC 1.7u 4.6 mm × 150 mm column (Waters) and an AQUITY UPLC PDA Detector (Waters Corporation, Milford, MA, United States) set to 220 nm. Runs were isocratic, and a 100 mM phosphate buffer (pH 6.8) was used as the mobile phase with 0.5 mL/min of flow rate, an injection volume of 2 μL, and a total run time of 15 min. The column temperature was set to 30°C for analysis. Bovine serum albumin (66,000 Da), cytochrome C (12,327 Da), aprotinin (6,512 Da), insulin A (2,531 Da), Leu-enkephalin (555.6 Da), Met-enkepalin (573.7 Da) Val-Tyr-Val (379.5 Da), and Gly-Tyr (238.2 Da) were used as standards. All were purchased from Merck. Chromatograms were manually integrated and separated into intervals of <0.2, 0.2–0.5, 0.5–1, 1–2, 2–5, and >5 kDa, expressed as percentages of the total area. All samples were analysed in triplicate.

### 2.6 Determination of FPH solubility

Protein extracts were prepared by dissolving 0.1 g of each FPH sample in 10 mL of distilled water. The solutions were homogenized and centrifuged. Water-soluble proteins were determined in triplicates by using the Lowry method (20). Bovine serum albumin (BSA) was used to prepare a standard curve. The absorbance of the incubated standards and samples was determined using a SpectraMax ix3 microplate reader (Molecular Devices; United States) at a wavelength of 750 nm. The protein solubility was calculated from the following formula (1):


(1) $$\mathrm{Solubility}\;\left(\%\right)=P_{s}/P_{t}\times 100$$

where P_s_ is soluble proteins and P_t_ is total protein content in the sample.

### 2.7 Degree of hydrolysis

The degree of hydrolysis (DH) was analysed by formol titration as the proportion (%) of free amino groups with regard to the total nitrogen in the sample previously determined by the Kjeldahl method (18).

A FPH sample of 1.5 g was weighed into a beaker and filled up to 50 g with distilled water. The pH was adjusted to 7.0 using 0.1 M NaOH and then 10 mL of 9% w/w formaldehyde with a pH of 8.5 was added into the beaker. The beaker was covered with aluminium foil and stirred for 5 min. For the titration, a TITROLINE 7000 automatic titrator (SI Analytics, Xylem Analytics Germany Sales GmbH & Co. KG, Germany), was used. The titrator was rinsed 3 times before starting the titration. Furthermore, the titration was set to pH 8.5 with stopping automatically when reaching a pH of 8.5. The samples were titrated with 0.1 M NaOH and the used amount of NaOH was recorded. Degree of hydrolysis was further determined as described by Kvangarsnes et al. (5).

### 2.8 Amino acid profile

About 50 mg of freeze-dried FPH was weighed into glass tubes, 1 mL 6 M HCl was added. The glass tubes placed into a heating cupboard for 24 h, at 105°C. Samples were diluted 50 times using distilled water before filtering through 0.22 μm.

For the derivatization, 200 μL of the sample were transferred to a glass tube, containing 600 μL 0.4 M borate buffer (pH 9). Four hundred microliters FMOC (9-fluorenylmethoxycarbonyl chloride, 15 mM in acetonitrile) was added, vortexed for one minute, and then allowed to stand at room temperature for 4 min. After amino acid derivatization with FMOC, 400 μL ADAM (60 mM in acetonitrile:water 2:1) was added.

Amino acids were analyzed using a Shimadzu Nexera XR HPLC system, equipped with a PDA detector (Shimadzu, United States). Separation of amino acids were carried out on a Restec ARC-18 column (10 mm × 2.1 mm) at 30°C. The mobile phase was 0.1% formic acid with 20 mM ammonium formate and 0.1% formic acid with 10 mM ammonium formate in 90:10 acetonitrile water in gradient mode, with a flowrate of 0.8 mL/min.

### 2.9 Protein oxidation

#### 2.9.1 Carbonyl groups

Carbonyl groups in the hydrolysate were determined using an immunoassay method developed by Buss et al. (21). The ELISA kit, STA-310 OxiSelect^™^ was purchased from Cell Biolabs, Inc. (San Diego, CA, United States). BSA standards (bovine serum albumin) and protein samples were adsorbed onto a 96-well plate at 4 degrees overnight. The protein carbonyls present in the sample were derivatized with DNP hydrazine and probed with an anti-DNP antibody, followed by an HRP conjugated secondary antibody. The carbonyl contents in the samples were calculated by comparison against a standard curve of commercial reduced and oxidized BSA standards provided in the kit and expressed as nanomoles of carbonyl per milligram of protein (nmol mg^−1^ protein).

#### 2.9.2 Thiol groups

Total thiol groups content was determined spectrophotometrically with Shimadzu UV-1800 UV/Visible Scanning Spectrophotometer (Shimadzu Europa GmbH, Germany) using the Ellman reagent (DTNB) according to the method previously described by Ellman (22) and Kvangarsnes et al. (5). To 100 μL of water soluble, and blanks (distilled water), 800 μL urea and 100 ul DTNB were added. Samples were mixed, incubated at room temperature for 30 min and centrifuged for 3 min at 11,300 g at room temperature. The absorbance was measured at 412 nm with a blank as reference. The thiol content was calculated using a molar extinction coefficient of 14,290 M^−1^ cm^−1^. The results are expressed as nmol/mg protein.

### 2.10 Colour measurements

Colour values of the FPH were measured using a Minolta Chromometer Model CR 400 (Konica Minolta, Japan) calibrated on a white reference plate before use. L* (lightness), a* (redness) and b* (yellowness) were measured on the protein hydrolysates in triplicate at room temperature. The L*, a*, and b* parameters of the CIELAB scale were measured according to the lab scale established by Commission Internationale de l’Éclairage (23).

### 2.11 Foaming properties

The foaming properties were determined in triplicate applying the method previously described (24). One percent FPH solution was prepared in distilled water. Aliquots of 50 mL (V1) were blended for 5 min using a magnetic stirrer (VELP Scientifica Srl, Usmate Velate, Italy) at the highest speed, poured into 250 mL graduated cylinders, and the volume of foam (V2) was immediately recorded at 0, 5, and 10 min. The foaming was calculated using the following equation: Foaming = (V2 − V1) × 100/V1. The foaming capacity was determined at 0 min and the foam stability (FS) after 5, and 10 min.

### 2.12 Turbidity measurement

The turbidity of samples was measured as described by Groleau et al. (25), with slight modification. Approximately 1% (w/v) of FPH sample was prepared in H_2_O at pH 2 to 12, the resulting mixture was vortexed and after standing for 30 min at room temperature, the absorbance was read at 500 nm (Synergy H1, Biotek, Bad Friedrichshall, Germany).

### 2.13 Intrinsic fluorescence spectroscopy

The intrinsic fluorescence spectrum of FPH sample was acquired applying a fluorescence spectrophotometer (Synergy H1, Biotek, Bad Friedrichshall, Germany). The sample was diluted in H_2_O to attain the same level of concentration of 0.5 mg/mL and were transferred in Greiner UV-Star^®^ 96 well plates with flat bottom clear cyclic-olefin copolymer (COC) wells. The excitation wavelength was set as 250–280 nm and the excitation and emission slit widths were set as 5 nm. The emission wavelength range was set up from 310 nm to 450 nm and the scanning speed was 10 nm/s.

### 2.14 Ultrafiltration of FPH

FPH samples were ultrafiltrated using a 3 kDa cut-off Millipore System UF ultrafiltration membrane (Millipore, Bedford, MA, United States) at 13,300 × g for 20 min, earlier in the biological activity analysis. After being dried in a Speed-Vac (Martin Christ Gefriertrocknungsanlagen GmbH, Osterode am Harz, Germany), the recovered solutions were kept at −80°C until use.

### 2.15 Determination of ferric reducing antioxidant power

The ferric reducing antioxidant power (FRAP) assay measures a sample’s capacity to convert ferrous ions (Fe^2+^) from ferric ions (Fe^3+^). With a small adjustment, the experiment was conducted using the previously described procedure (26). Consequently, 140 μL of FRAP reagent (1.3 mL of a 10 mM TPTZ solution in 40 mM HCl, 1.3 mL of 20 mM FeCl_3_ × 6 H_2_O, and 13 mL of 0.3 M acetate buffer) was combined with 10 μL of FPH solutions at the final concentrations of 0.1, 0.5, and 1.0 mg/mL. Using a Synergy H1 microplate reader, the absorbance was measured at 595 nm following a 30 min incubation period at 37°C.

### 2.16 *In vitro* scavenging DPPH radical assay

The DPPH assay was used to measure the antioxidant activity (7). Fifteen microliters FPH solutions were mixed with 45 μL DPPH methanolic solution (at final concentration of 0.1, 1.0 and 5.0 mg/mL). The mixture was incubated at RT for 30 min in the dark. The Synergy H1 microplate reader was used to measure the absorbance at 520 nm.

### 2.17 *In vitro* scavenging ABTS radical assay

The assay based on the reduction of the ABTS radical induced by antioxidants, was performed according to the method (27). In order to make the ABTS^●+^ preparations, 2.45 mM potassium persulfate and 7 mM ABTS solution were combined (1:1) and left at RT in the dark for 16 h. To make the ABTS reagent, the ABTS^●+^ was diluted in 5 mM phosphate buffer (pH 7.4) until a steady absorbance of 0.700 (±0.02) at 730 nm was obtained. For the assay, 10 μL of FPH at the final concentrations of 0.05, 0.1, 0.5, 1 and 2.5 mg/mL were added to 140 μL of the diluted ABTS^●+^. The microplate was incubated for 30 min at 30°C, and the absorbance was read at 730 nm applying Synergy H1. A Trolox calibration curve (60–320 μM) was used to evaluate the TEAC values.

### 2.18 Cell culture

Caco-2 cells were obtained from INSERM (Paris, France) and cultured in DMEM with 25 mM of glucose, 3.7 g/L of NaHCO_3_, 4 mM of stable L-glutamine, 1% nonessential amino acids, 100 U/L of penicillin, and 100 μg/L of streptomycin (complete medium), 10% heat-inactivated FBS at 37°C in a 90% air/10% CO_2_ atmosphere.

### 2.19 MTT (3-(4,5-dimethylthiazol-2-yl)-2,5-diphenyltetrazolium bromide) assay

Caco-2 cells (3 × 10^4^) were seeded in a 96 well plate and treated with FPH (0.1 to 10 mg/mL) or vehicle (H_2_O) in complete DMEM for 48 h at 37°C under a 5% CO_2_ atmosphere. Eventually, the treatment was eliminated a filtered solution containing 100 μL of MTT (0.5 mg/mL) was added. Following a 2 h incubation period at 37°C with 5% CO_2_ atmosphere, the MTT solution was removed and 100 μL of the lysis buffer (8 mM HCl + 0.5% NP-40 in DMSO) was added to each well. The absorbance at 575 nm was measured using the Synergy H1 microplate reader (Biotek, Bad Friedrichshall, Germany) after 10 min of gentle shaking.

### 2.20 Measurement of intracellular ROS

3 × 10^4^ Caco-2 cells/well were seeded on a black 96-well plate overnight in growth medium. The next day, the medium was discarded and replaced with 50 μL/well of fresh medium and 50 μL/well of master reaction mix were added and the plate was incubated at 5% CO_2_ 37°C for 1 h in the dark. Cells were treated with 10 μL of 11x FPH to reach the final concentrations of 2.5 mg/mL at 37°C in the dark for 24 h. Cells were treated with H_2_O_2_ at a final concentration of 1 mM to induce ROS for 30 min at 37°C in the dark, and fluorescence intensity (ex./em. 490/525 nm) was measured using a Synergy H1 microplate reader.

### 2.21 Lipid peroxidation malondialdehyde assay

2.5 × 10^5^ cells/well Caco-2 cells were seeded in a 24-well plate, and the next day treated with 2.5 mg/mL of FPH for 24 h at 37°C under a 5% CO_2_ atmosphere. Then, cells were incubated with H_2_O_2_ 1 mM or vehicle (H_2_O) for 30 min and lysed following the manufacturer’s protocol. For analysis, 100 μL of each reaction mixture was pipetted into a 96-well plate, and the absorbance at 532 nm was measured by a Synergy H1 microplate reader.

### 2.22 Statistical analysis

All results were expressed as the mean ± standard deviation (s.d.), where *p*-values <0.05 were considered to be significant. Statistical analyses were performed by one-way and two-way ANOVA followed by Tukey’s post-test (GraphPad Prism 9, GraphPad Software, La Jolla, CA, United States). *T*-test was used to compare the control sample and samples treated with ultrasound (Stata v18.0, StataCorp).

## 3 Results and discussion

### 3.1 Proximate composition of FPH

The proximate composition of mackerel FPH is shown in Table 1.

**Table 1:** Proximate composition of PFH obtained from Atlantic mackerel.

Composition	Amount, g/100 g
Protein content	81.0 ± 0.8
Water content	5.8 ± 0.7
Ash	9.4 ± 0.2
Fat	3.8 ± 0.0

### 3.2 Molecular weight distribution

**Figure 1:**
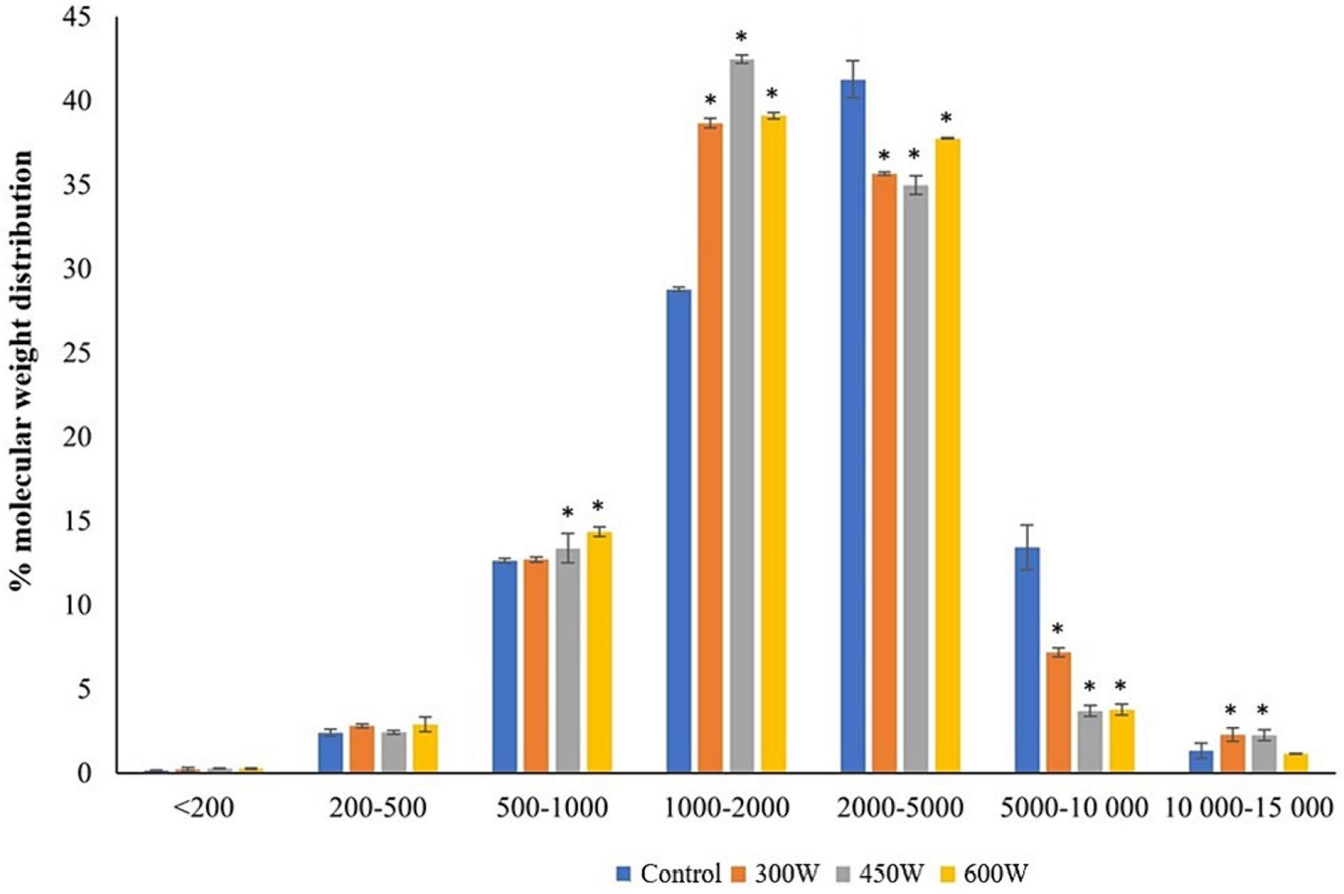
Molecular weight distribution in PFH obtained from Atlantic mackerel. All the data sets have been analyzed by one-way ANOVA. (*) *p* < 0.05.

The molecular weight distribution (MWD) of mackerel FPH (Figure 1) revealed that ultrasound treatment caused a significant (*p* < 0.05) decrease in particle size for peptides with MW between 2,000 and 10,000 Da with a corresponding significant increase in particle size for the peptides with MW 1,000–2,000 Da. Similar results were observed in previous studies investigating the effect of ultrasonication with a 20 kHz probe at higher intensities on MW distribution of whey proteins (28). They have shown that ultrasound treatment can significantly change the structural properties of peptides through thermal denaturation, fragmentation, and rearrangement of peptide chains by breaking hydrogen bonds and disrupting secondary structures (28). It was also shown that ultrasound can increase the specific free surface area and decrease the particle size of proteins and peptides by creating micro-holes and cavities in their surfaces (28). Therefore, we hypothesize that the ultrasound-induced changes in molecular size distribution of FPH are associated with partial denaturation of larger peptides and cleavage of intermolecular hydrophobic interactions, resulting in particle size reduction and increase in amount of small bioactive peptides.

### 3.3 Solubility of FPH

**Figure 2:**
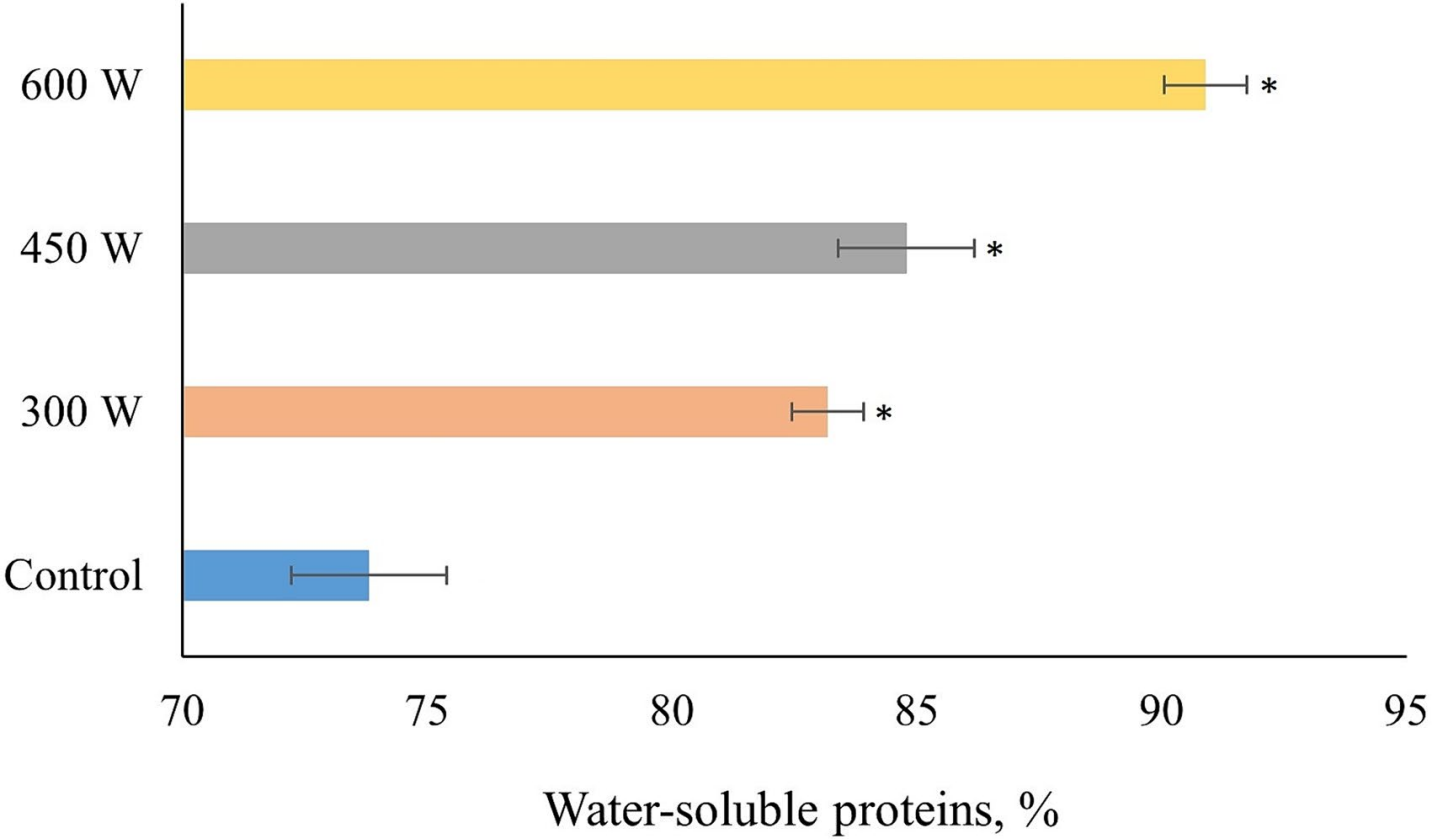
Protein solubility of PFH obtained from Atlantic mackerel. All the data sets have been analyzed by one-way ANOVA. (*) *p* < 0.05.

According to the results shown in Figure 2, there is a gradual significant (*p* < 0.05) increase in protein solubility for all US-treated FPH compared to control samples. The linear increase in solubility of FPH after US-treatment can be explained by the changes in molecular weight of peptides with increasing intensity of the ultrasound treatment. In previous studies it was shown that ultrasound treatment can increase the solubility range of fish protein hydrolysates from 5 to 10 kDa to 20 to 30 kDa (29, 30). This is because ultrasound treatment can cause denaturation or unfolding of proteins, which reduces their secondary and tertiary structure, while increasing their flexibility. This allows more peptides to be generated from a given amount of protein by enzymatic hydrolysis (31). This statement is supported by the significant correlation (*R* = 0.999, *p* < 0.05) between the amount of small peptides (500–1,000 Da) generated after ultrasonication and protein solubility. The linear increase in the amount of small peptides with a molecular size of 500–1,000 Da correlating with the increase in protein solubility, may be explained by the deagglomeration effects of US-treatments observed in other studies (32, 33). Thus, the ultrasonication was breaking down the insoluble peptide aggregates present in FPH leading to the release of small soluble peptides by rupturing non-covalent interactions, which can be seen from the linear increase in the protein solubility after the sonication (32).

### 3.4 Degree of hydrolysis of mackerel hydrolysates

**Figure 3:**
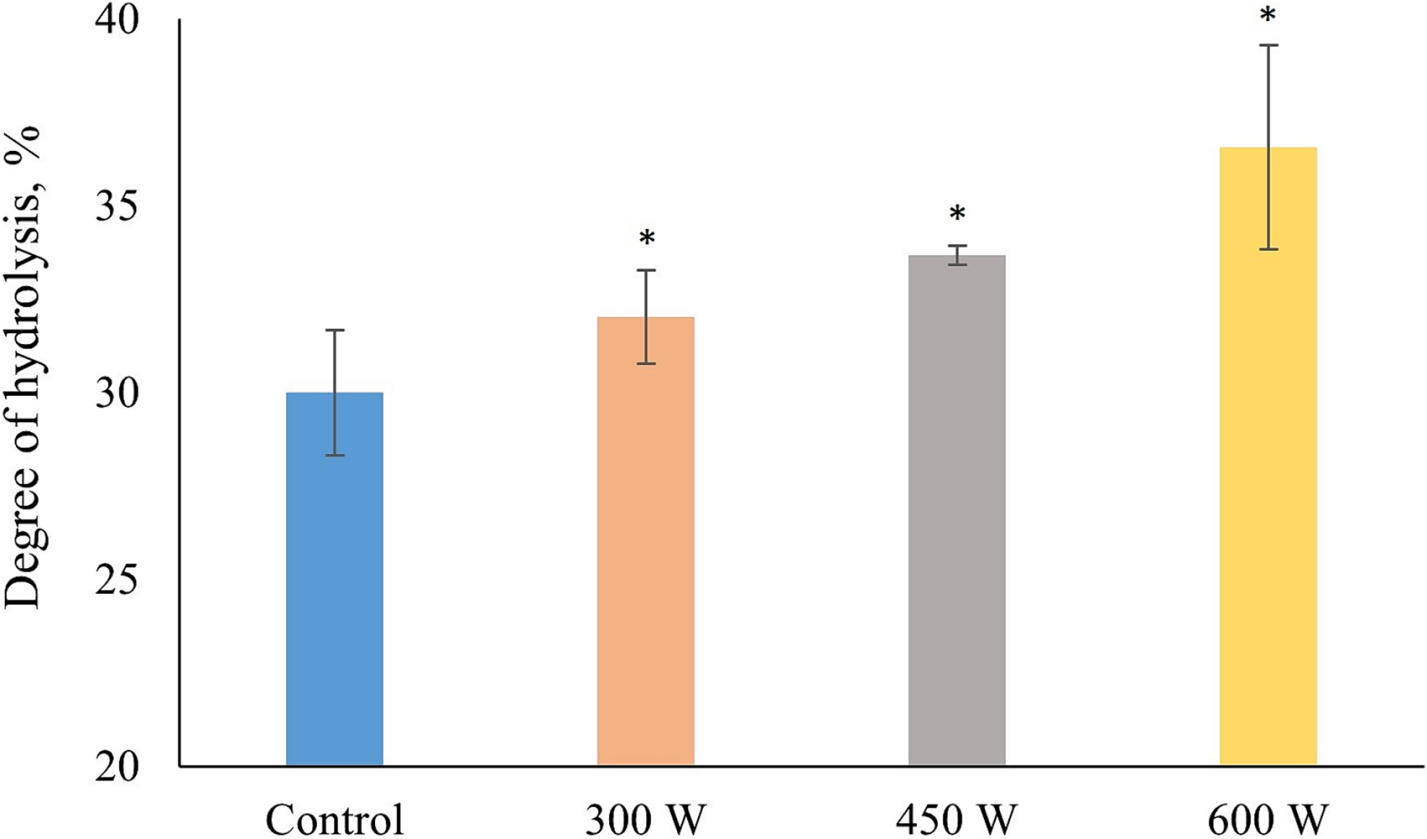
Degree of hydrolysis in PFH obtained from Atlantic mackerel. All the data sets have been analyzed by one-way ANOVA. (*) *p* < 0.05.

Degree of hydrolysis increased significantly (*p* < 0.05) in all ultrasound-treated samples compared to control (Figure 3). In addition, a linear trend of DH increase which significantly correlated with the protein solubility of FPH (*R* = 0.996, *p* < 0.05) was observed. This phenomenon can be explained by the cavitation effect. Ultrasound treatment creates cavitation bubbles in the liquid medium of FPH solution, which are regions of low pressure and high density. These bubbles can collapse and generate shock waves, which can cause denaturation or unfolding of proteins or peptides, and also break down the peptide molecules into smaller pieces (34), increasing the amount of free amino acid groups and degree of hydrolysis.

### 3.5 Total amino acid profile

The total amino acid profile of mackerel FPH is displayed in Table 2.

**Table 2:** Amino acid composition in FPH, % of total amino acids.

Amino acid	Control	300 W	450 W	600 W
Arginine	3.68 ± 0.32	4.04 ± 0.8	4.44 ± 0.02	3.61 ± 0.48
Serine/aspartic acid	9.07 ± 1.06	9.88 ± 2	11.58 ± 0.03	9.3 ± 0.52
Glutamic acid	8.65 ± 0.47	9.38 ± 1.29	11.14 ± 0.58*	9.29 ± 2.36
Threonine	3.69 ± 0.14	4.17 ± 0.31	4.75 ± 0.3*	4.62 ± 0.07*
Glycine	15.21 ± 0.51	14.77 ± 0.58	15.11 ± 2.68	15.82 ± 2.02
Alanine	8.4 ± 0.48	6.89 ± 0.17	7.86 ± 0.38	7.65 ± 0.34
Proline	9.69 ± 0.3	8 ± 0.9	4.59 ± 1.97	6.86 ± 0.02*
Valine	0.27 ± 0.02	0.72 ± 0.79	0.01 ± 0.01*	0.2 ± 0.09
Phenylalanine	2.43 ± 0	2.19 ± 1.03	3.81 ± 0.39*	2.74 ± 0.36
Isoleucine	3.26 ± 0.04	3.45 ± 0.44	3.68 ± 0.22	3.35 ± 0.26
Leucine	12.37 ± 0.04	13.05 ± 1.66	9.5 ± 1.18	13.41 ± 2.6
Cystine	0.68 ± 0.21	2.43 ± 0.27*	3.28 ± 0.26*	2.83 ± 0.78*
Histidine/cysteine/tyrosine	22.6 ± 0.07	20.95 ± 0.9	20.17 ± 2.17	20.21 ± 2.4

Mean values ± standard deviation is shown. (*) *p* < 0.05.

The most abundant amino acid was Glycine, and this is due to the high content of skin in the raw material. The highest proportion was found in the sample treated with ultrasound with a power of 600 W (15.82). Glycine is a hydrophobic amino acid, and Rajapakse et al. (35) stated that this amino acid is highly soluble in lipids and can have more capability to gain closer access to radicals compared to neutral and hydrophilic amino acids. Phenylalanine, tyrosine, isoleucine, proline and histidine has been described as a source of bitter taste from peptides (36). FPH treated with US with a power of 450 W has a significantly higher content of phenylalanine, compared to control. The proportion of isoleucine is also highest in the ultrasound treated sample (450 W), but the difference is not significant. The higher content of these amino acids in the peptides may cause a more bitter FPH if these are not stable under storage and generate more free amino acids. However, no sensory analysis was carried out during this study.

### 3.6 Protein oxidation

The results of protein oxidation of FPH extracted from Atlantic mackerel assessed in terms of total carbonyls and thiol groups, are shown in Table 3.

**Table 3:** Protein oxidation parameters of PFH obtained from Atlantic mackerel.

Protein oxidation parameter	Control	300 W	450 W	600 W
SH-groups (nmol thiol/mg protein)	16.04 ± 0.43	11.88 ± 0.33*	14.68 ± 0.31*	15.74 ± 0.39
Carbonyl groups (nmol/mg protein)	3.08 ± 0.06	3.27 ± 0.15	4.13 ± 0.26	4.19 ± 0.83

(*) *p* < 0.05.

There was a small increase in total carbonyls accompanied by a small decrease in thiol groups in all US-treated samples compared to control. However, the changes were significant (*p* < 0.05) only for the samples treated with 450 W and 600 W in terms of total carbonyls, and for the samples treated with 300 W and 450 W in terms of thiol groups. This phenomenon can be explained by the ultrasound-induced cavitation causing the unfolding of tertiary and secondary structures of protein molecules. These changes can lead to the exposure of amino acid residues that are prone to oxidation to the surrounding environment, thereby reducing the total thiol groups and increasing the total protein carbonyls (37). In addition, the cavitation effect of ultrasound can lead to the formation of reactive oxygen species (ROS) in the amount of fish oil (3.8% in the present study) that is normally found in FPH after enzymatic hydrolysis (37). The structural changes caused by ultrasound treatment may increase the accessibility of peptides to ROS due to the increased contact area between the peptides and oxidized lipids, leading to the formation of carbonyl groups in FPH. Moreover, the energy input from the ultrasonication can provide the necessary activation energy for the oxidation reactions to occur. The reaction between peptide molecules and reactive species formed as a result of the ultrasound cavitation effect may also result in the formation of disulfide bonds. This process effectively reduces the number of free thiol groups as they are converted into disulfide bonds (38).

It is highly important for FPHs to have low values of protein oxidation, since it can affect their functional properties. Protein oxidation induced by ultrasound treatment can significantly impact the functional properties of FPHs in several ways, including alterations in the secondary structure of protein molecules, resulting in the reduction of the α-helix content and the increase the β-sheet content, which can affect the solubility and stability of the protein hydrolysates (39). However, in the present study, even though the changes in protein oxidation parameters were significant for some US-treated samples compared to control, the results show low values of protein oxidation for all FPH samples.

### 3.7 Color parameters

The consumer acceptability and commercialization of protein hydrolysates primarily depend on their sensory attributes, including color parameters. According to previous studies, lighter-colored fish protein hydrolysates are often associated with freshness and purity and are therefore perceived as having higher quality compared to darker FPHs (40). The effects of different ultrasound post-treatments on the color parameters of FPH extracted from Atlantic mackerel are presented in Table 4.

**Table 4:** Color parameters of PFH obtained from Atlantic mackerel.

	Control	300 W	450 W	600 W
L*-value	71.40 ± 1.53	79.70 ± 0.79*	79.46 ± 0.56*	80.14 ± 0.11*
a*-value	1.92 ± 0.22	−0.40 ± 0.10*	−0.47 ± 0.04*	−0.40 ± 0.04*
b*-value	22.39 ± 0.63	17.82 ± 0.95*	17.16 ± 0.04*	17.05 ± 0.14*

(*) *p* < 0.05.

According to the results displayed in Table 4, all FPHs subjected to ultrasound post-treatment had significantly (*p* < 0.05) higher L*-values and significantly lower a*-values and b*-values compared to control. These results show that FPH became much lighter and less yellowish and reddish after the ultrasound treatment, that can positively influence their perception by consumers and further commercialization. This phenomenon is probably due to the modification of the secondary structure of protein molecules as the effect of ultrasound treatment, during which the absorption peaks of the light can shift to higher wavelengths, thereby influencing the color of FPH (39).

In the present study, all FPHs subjected to ultrasound treatment are more likely to be attractive to consumers due to lighter appearance and lower redness and yellowness, which can be associated with freshness, better quality, and lower oxidation values compared to control sample, and thus positively influence their marketability.

### 3.8 Foaming and turbidity properties

**Figure 4:**
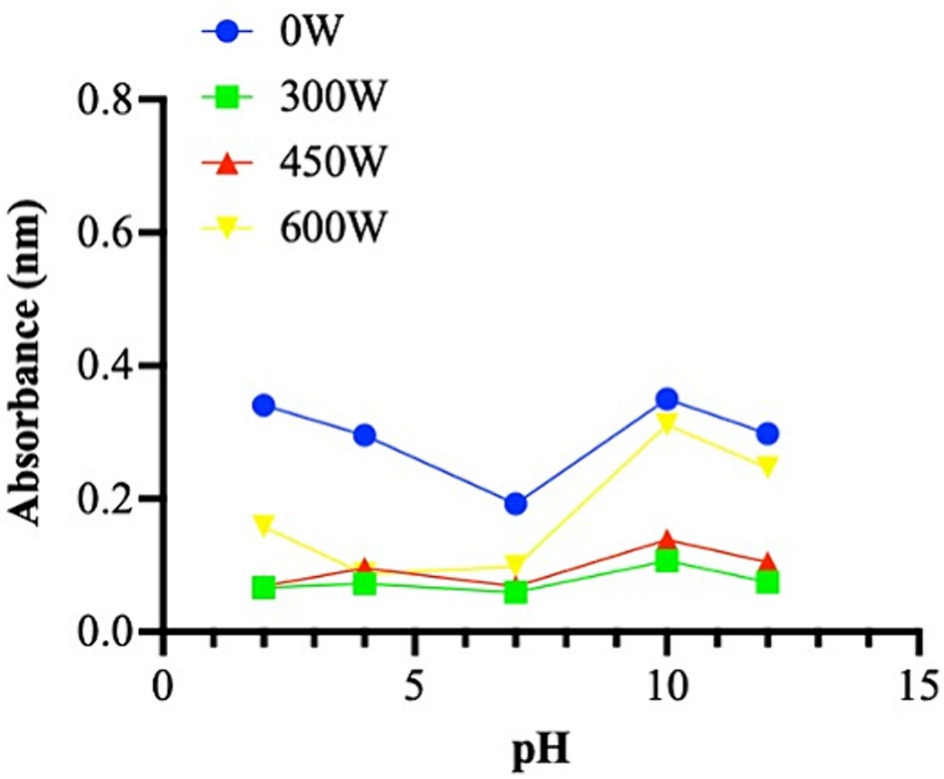
Turbidity property presented as absorbance (nm) in different pH (from 2 to 12). The data are represented as the means of three independent experiments, performed in duplicate.

Numerous studies reported in the literature indicate that fish hydrolysates have good foaming properties (41, 42), however no foaming capacity was found in any of mackerel hydrolysate samples in this study. One method of looking for potential peptide aggregation forms in hydrolysates is turbidity (43). Turbidity changes depending on the ultrasound treatment applied. In particular, the untreated sample appears to have a higher turbidity than the ultrasonicated samples. The pH-dependency of turbidity for the hydrolysates is quite similar. In the sample without treatment, there is an apparent decrease in turbidity from pH 2 to 7, and an increase from pH 7 to pH 12 (Figure 4). The samples subject to 300 W and 450 W ultrasound treatment show a similar trend, while the subjected to the highest ultrasound potency displayed a greater aggregation state at pH 10 to 12.

### 3.9 Intrinsic fluorescence of FPH

**Figure 5:**
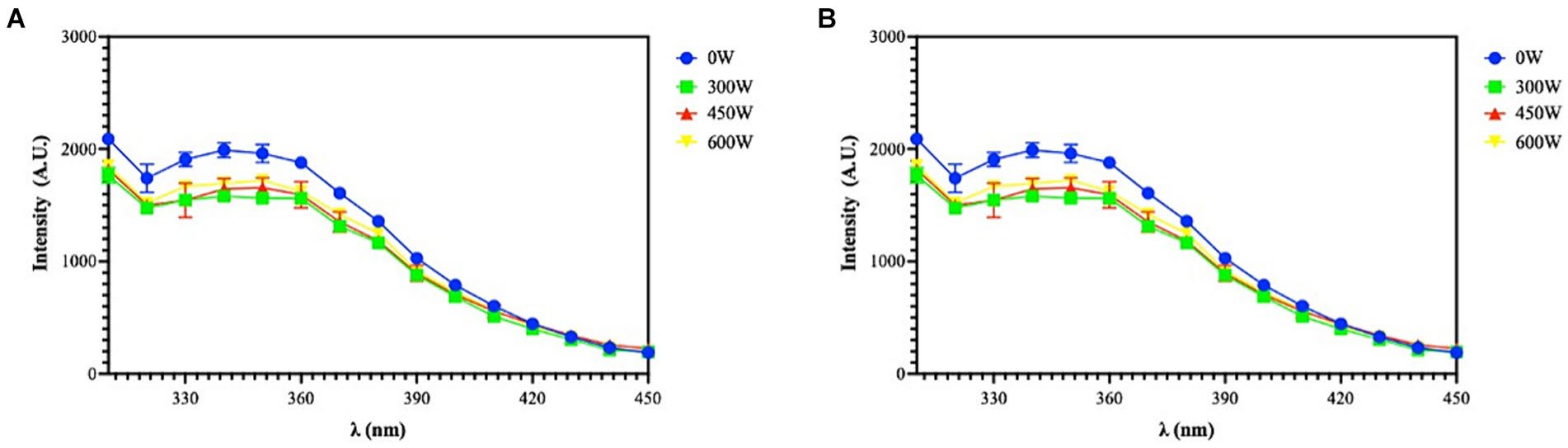
Intrinsic fluorescence signal detection of FPH at 250 nm **(A)** and 280 nm **(B)** excitation. The data are represented as the means of two independent experiments, performed in duplicate.

The fluorescence resulting from the presence of tryptophan (Trp), tyrosine (Tyr), and phenylalanine (Phe), which are excited at 250 and 280 nm, was detected using the intrinsic fluorescence spectroscopy technique. The chromophores are exposed to an aqueous environment, as seen by the peaks at 310 and 340 nm, with 250 nm excitation, and at 310 and 350 nm, with 280 nm excitation, which demonstrate a shift towards a longer emission wavelength (Figure 5).

### 3.10 Biochemical characterization of the biological activities of FPH

#### 3.10.1 *In vitro* antioxidant activity

**Figure 6:**
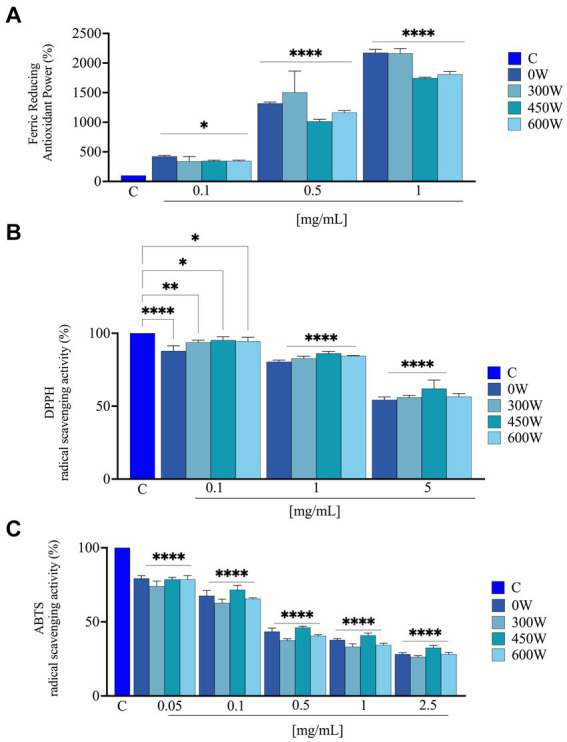
Direct radical scavenging activity of FPH samples treated with different ultrasound power. Antioxidant power evaluation by ferric reducing antioxidant power (FRAP) **(A)**; by DPPH radical scavenging activity **(B)** and by ABTS assay **(C)**. Data represent the mean ± s.d. of three independent experiments, and each experiment was performed in triplicate. C, control sample. All the data sets have been analyzed by one-way ANOVA. (*) *p* < 0.05, (**) *p* < 0.01, (****), and *p* < 0.0001.

The hydrolysis conditions and pre-treatments had a significant impact on the release of antioxidant peptides (44). As indicated in Figure 6, FRAP, DPPH and ABTS assays were used to assess the antioxidant activity of FPH samples. The *in vitro* FRAP was investigated in the range of 0.1–1 mg/mL, the *in vitro* DPPH assays was assessed in the range of 0.1–5 mg/mL and the *in vitro* ABTS assay was examined in the range of 0.05–2.5 mg/mL. Results showed that FPH 0 W, 300 W, 450 W and 600 W increased FRAP by 422.7 ± 17.4%, 340.9 ± 80.5%, 345.5 ± 14.8% and 345.5 ± 14.8% at 0.1 mg/mL, compared to vehicle, respectively. For 0.5 mg/mL the levels were increased by 1318.1 ± 23.5%, 1505.0 ± 385.6%, 1013.6 ± 34.4% and 1163.6 ± 36.4%, compared to vehicle. Finally, at 1 mg/mL the FRAP were increased by 2172.7 ± 56.5%, 2163.6 ± 82.6%, 1745.4 ± 14.8% and 1809.1 ± 48.1%, compared to vehicle, respectively (Figure 6). Interestingly, at 0.5 and 1 mg/mL the FPH treated with 450 W and 600 W displayed a slight loss of the antioxidant activity compared to untreated and 300 W samples, suggesting that the stronger ultrasound treatment does not provide an improvement in the ferric reducing capacity of the hydrolysates. Findings indicated that FPH 0 W, 300 W, 450 W and 600 W reduced the DPPH radical up to 54.3 ± 2.0%, 56.0 ± 1.4%, 62.0 ± 5.8%, and 56.5 ± 2.1%, compared to vehicle, respectively, at the highest tested concentration of 5.0 mg/mL (Figure 6). In detail, at 0.1 mg/mL DPPH radical was reduced by 87.8 ± 3.5%, 93.7 ± 1.5%, 95.2 ± 2.3%, and 94.5 ± 2.8%, by FPH 0 W, 300 W, 450 W and 600 W, respectively. FPH 0 W, 300 W, 450 W and 600 W decrease the DPPH levels by 80.4 ± 1.1%, 82.6 ± 1.5%, 86.2 ± 1.4% and 84.4 ± 0.4%, respectively, at 1.0 mg/mL, and by 54.3 ± 2.0%, 56.0 ± 1.4%, 62.0 ± 5.8% and 56.5 ± 2.1%, respectively, at 5.0 mg/mL (Figure 6). As indicated in Figure 6, FPH 0 W, 300 W, 450 W and 600 W scavenged the ABTS radical by 71.8 ± 1.0%, 73.7 ± 0.8%, 67.5 ± 1.6% and 71.8 ± 1.2%, compared to vehicle, respectively, at the highest assayed concentration of 2.5 mg/mL. More in detail, FPH 0 W, 300 W, 450 W and 600 W scavenged the ABTS radical by 20.7 ± 1.8%, 26.0 ± 3.5%, 21.4 ± 1.4% and 21.4 ± 2.5% at 0.05 mg/mL and by 32.5 ± 3.6%, 37.3 ± 2.5%, 28.4 ± 2.9% and 34.4 ± 0.5% at 0.1 mg/mL, respectively. The same samples scavenged the ABTS radical by 62.3 ± 0.8%, 66.8 ± 1.9%, 59.2 ± 1.6%, and 65.6 ± 1.2% at 1 mg/mL, and at 2.5 mg/mL by 71.8 ± 1.0%, 73.7 ± 0.8%, 67.5 ± 1.6% and 71.8 ± 1.2%, respectively. A dose-dependent response was observed in all the antioxidant assays. Thus, FPH samples, both untreated and ultrasound-treated have shown the ability to scavenge DPPH and ABTS radicals and to reduce iron with a concentration-dependent trend, and in a significant way compared to vehicle, demonstrating to possess different antioxidant mechanisms of action. In particular, all the samples, treated with different ultrasound power, showed comparable bioactivity for ABTS and DPPH assays, while a slight reduction in FRAP activity was noticed for samples treated with the highest ultrasound power at 0.5 and 1 mg/mL. These findings suggest that fish hydrolysates’ *in vitro* antioxidant activity and *in vitro* mechanism of action can depend both on the fish matrix starting material and on the ultrasound treatment of the final product. The results are in line with what has been reported in several different protein hydrolysates obtained from different types of fish such as stone fish (*Actinopyga lecanora*) (45), croaker (*Otolithes ruber*) (46) and carp (*Cyprinus carpio*) (47).

#### 3.10.2 *In vitro* DPP-IV inhibitory activity

**Figure 7:**
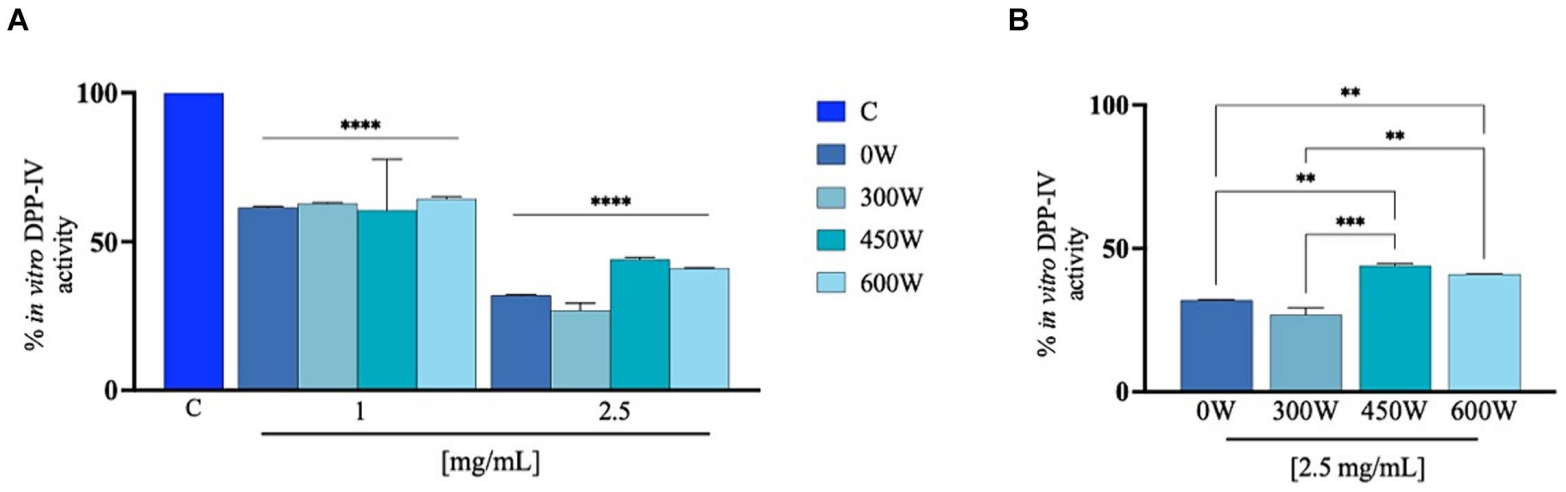
Cell-free inhibition of the activity of human recombinant DPP-IV **(A)** and comparison between treatment at 2.5 mg/mL **(B)**. The data are represented as the means ± s.d. of six independent experiments, performed in triplicate. Statistical analysis was performed by one-way ANOVA, followed by Tukey’s post-hoc test (****) *p* < 0.0001.

The ability of FPH to influence DPP-IV activity was assessed using preliminary biochemical investigations using human purified recombinant DPP-IV enzyme. The enzyme was incubated with the FPH at the concentration of 1 and 2.5 mg/mL. Figure 7 shows that the FPH 0 W, 300 W, 450 W and 600 W reduces DPP-IV activity *in vitro* by 38.6 ± 0.5%, 37.1 ± 0.2%, 39.5 ± 17.0% and 35.6 ± 0.7% at 1 mg/mL and by 68.1 ± 0.2%, 73.11 ± 2.4%, 55.9 ± 0.7% and 58.9 ± 0.2% at 2.5 mg/mL, compared to vehicle, respectively. In line with the FRAP results, a drop in the DPP-IV inhibitory activity was observed at the highest tested concentration (2.5 mg/mL) for 450 W and 600 W FPH, as reported in Figure 7B. In particular, as shown in the graph, the *in vitro* DPP-IV inhibitory activity is significantly reduced for 450 W and 600 W FPH samples compared to untreated (0 W) and 300 W FPH at 2.5 mg/mL, while 0 W and 300 W samples displayed a comparable DPP-IV inhibitory bioactivity. These results are in line with literature evidence suggesting that a significant increase in ultrasonic power applied to discarded cowhide collagen led to peptides with reduced DPP-IV inhibitory activity (48). It has been shown that short ultrasound treatment allows the release of a higher number of bioactive peptides and therefore the identification of an adequate ultrasound treatment can facilitate the cleavage of proteins, and further ultrasound treatment could cause the development of aggregates (49). Using *in vitro* assays, the antioxidant and DPP-IV inhibitory activities of FPH were screened. However, to effectively utilize this potential ingredient in managing chronic diseases, such as diabetes—where both oxidative stress and DPP-IV inhibition can be targeted—it is essential to conduct more in-depth *in vivo* studies using animal models. While *in vitro* studies are valuable for screening potential bioactive peptide mixtures with multifunctional properties, demonstrating the therapeutic efficacy of these peptides requires animal studies that better reflect real physiological conditions. This additional research will provide crucial evidence to support the application of FPH in clinical settings.

### 3.11 Characterization of biological activities of FPH at cellular levels

#### 3.11.1 Effect of FPH on the Caco-2 cell vitality

**Figure 8:**
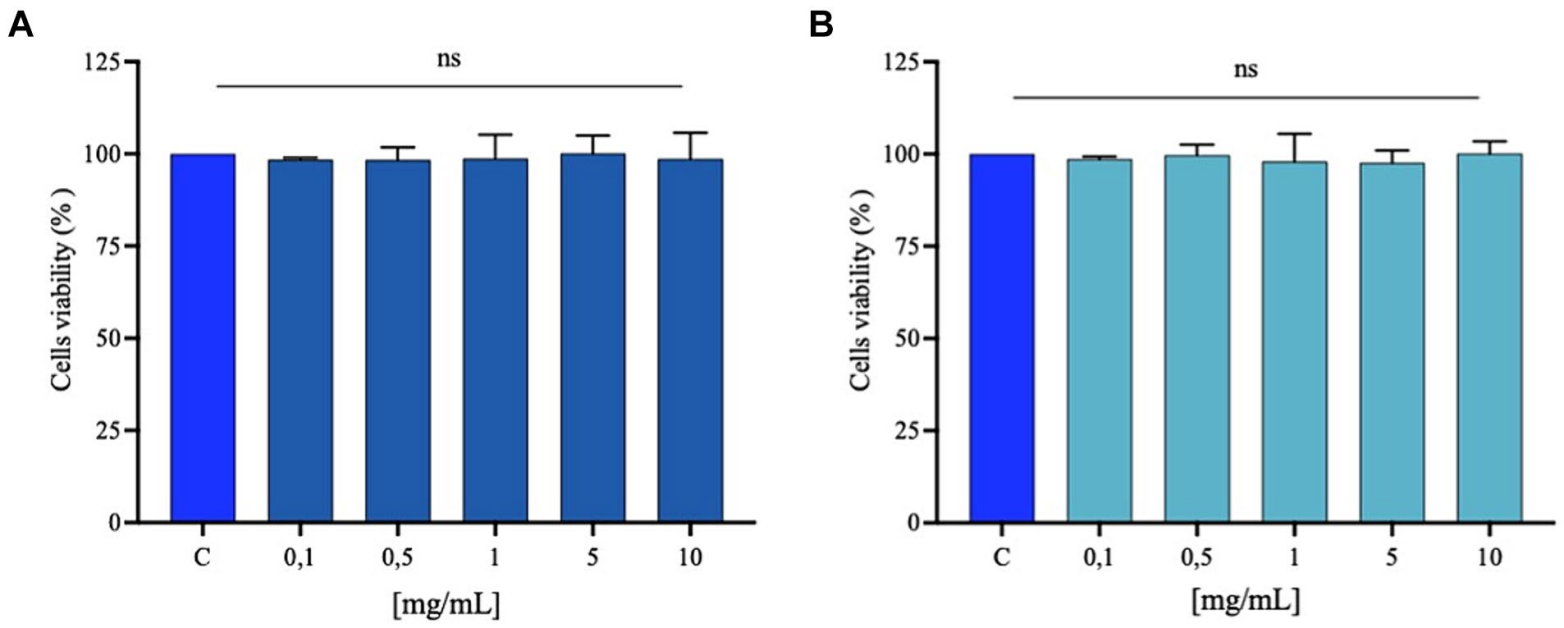
Caco-2 cell viability after FPH treatment with 0 W **(A)** and 300 W **(B)**. Bar graphs indicating the results of cell viability assay of Caco-2 cells after FPH (0.1–10 mg/mL) treatment for 48 h. The data points represent the averages ± SD of three experiments in triplicate, statistical analysis was performed by one-way ANOVA. C, control sample (H_2_O), ns, not significant.

Since no increase in biological activity related to the increase in ultrasound power was observed in the preliminary *in vitro* studies, the experiments at the cellular level were conducted by testing the samples without treatment (0 W) and the sample treated with the lower power (300 W). Experiments on cellular viability were carried out to exclude any possible harmful effects of the FPH treatment on the Caco-2 cell line. Following a 48 h treatment, no cytotoxic effect was shown up to 10 mg/mL in comparison to control cells (only treated with vehicle), suggesting that FPH did not affect Caco-2 cells cytotoxically in this dose range (Figure 8).

#### 3.11.2 FPH decrease the H_2_O_2_-induced ROS and lipid peroxidation levels in Caco-2 cells

**Figure 9:**
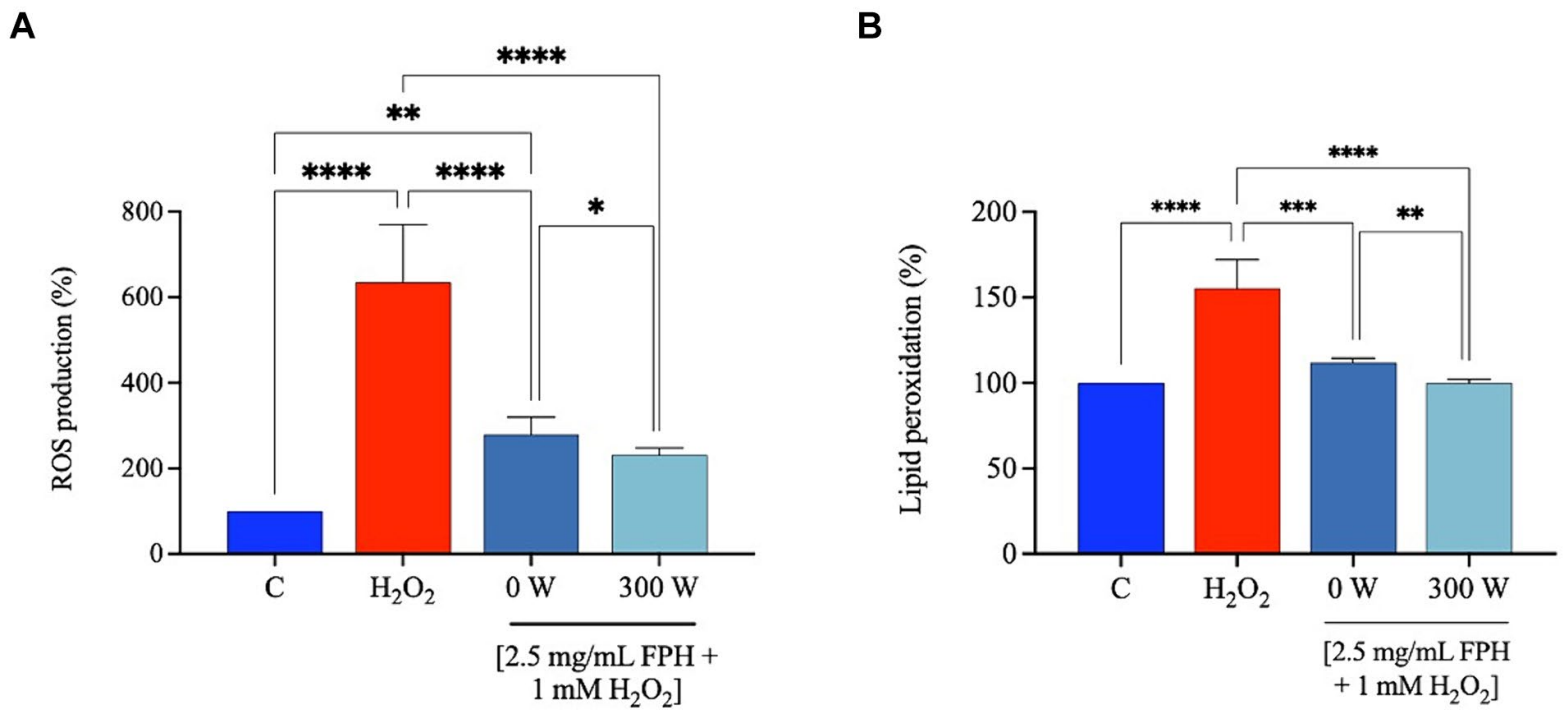
Effect of FPH on the modulation of intracellular ROS levels **(A)** and lipid peroxidation **(B)** after H_2_O_2_-oxidative stress induction (1 mM). The data are represented as the means ± SD of three independent experiments, performed in triplicate. Statistical analysis was performed by one-way ANOVA, followed by Tukey’s post-hoc test and by unpaired *t*-test, (*) *p* < 0.05, (**) *p* < 0.01, and (****) *p* < 0.0001.

It has been reported that marine bioactive peptides from a variety of marine organisms act as antioxidants by chelating pro-oxidant metal ions (50), minimizing reactive oxygen species (ROS) production (51), and reducing lipid peroxidation (52). Considering data from the preceding assays, which demonstrated a decline in both the *in vitro* DPP-IV inhibitory activity and *in vitro* FRAP bioactivity for the FPH samples post-treated with the highest power of ultrasound (450 W and 600 W) when tested at higher concentrations, only 0 W and 300 W were selected for a more thorough evaluation of the antioxidant qualities at the cellular level, assessing their protective effects following the production of oxidative stress using H_2_O_2_ on human intestinal Caco-2 cells. Elevated levels of ROS within cells can oxidize and damage important biological macromolecules, including cytomembranes, and are a significant factor in the etiology of numerous human disorders (53). Cell membrane lipids are vulnerable to oxidative stress induced from ROS. This can trigger a well-defined chain reaction that produces end products such malondialdehyde (MDA) and other related molecules. Figure 9A shows that the treatment of Caco-2 cells with H_2_O_2_ alone produces a significant increase of intracellular ROS levels, compared to standard conditions (vehicle), by 634.7 ± 134.1%, which was attenuated by the treatment with FPH 0 W and 300 W by 279.1 ± 39.7% and 231.4 ± 16.1% at 2.5 mg/mL. These findings indicate that both samples protect against the H_2_O_2_-induced oxidative stress and in addition, the US treatment (300 W) further significantly improves the antioxidant capacity of the hydrolysate as reported on the graph (Figure 9). In line with these results both hydrolysates were able to significant ameliorate the lipid peroxidation on Caco-2 cells after H_2_O_2_ stimulation. In detail, the H_2_O_2_ (1 mM) treatment increased the MDA levels up to 155.2 ± 17.0% compared to control conditions (vehicle). The pre-treatment of Caco-2 cells with both hydrolysates caused a significant reduction of lipid peroxidation restoring basal conditions. Figure 9B clearly shows that 0 W decreases the lipid peroxidation up to 111.9 ± 2.6% and 300 W up to 100.0 ± 2.3% at 2.5 mg/mL, further confirming that the US treatment (300 W) can significantly ameliorate the FPH antioxidant power at cellular level compared to untreated FPH (0 W). The ultrasonic power, equipment, and protein source have a major role in the effectiveness of ultrasound application during pretreatment or enzymatic hydrolysis, and in this case, and the FPH antioxidant activity is enhanced by the treatment.

## 4 Conclusion

Considering all the results obtained in our study, FPH have significant potential in the nutraceutical and food industries due to their rich composition of bioactive peptides, which offer various health benefits. In the nutraceutical sector, FPH can be incorporated into dietary supplements aimed at managing oxidative stress and diabetes promoting cardiovascular health. In the food industry, FPH can serve as functional ingredients to improve nutritional properties and texture in products such as protein-rich snacks, soups, and beverages. Additionally, FPH can improve the bioavailability of other nutrients and act as a natural preservative, which aligns with the growing consumer demand for clean-label products. Their versatility, along with their potential to promote health and well-being, positions FPH as a promising component in the development of innovative food and nutraceutical solutions. Ultrasonication may offer many benefits to FPH, including the improvement of overall quality and techno-functional characteristics of the final ingredient. This study has shown that ultrasound post-treatment significantly increased protein solubility and degree of hydrolysis of all US-treated samples compared to control through reduction of particle size of peptides, thereby enhancing the functionality of FPH. Furthermore, the structural changes induced in protein molecules by ultrasonic acoustic cavitation improved color parameters of the FPH, notably lightness, which may further positively influence the perception of this ingredient by consumers. Use of ultrasound on hydrolysates may cause a hydrolysate with a more bitter taste, on the other hand, use of ultrasound may liberate free amino acids with antioxidative functions. Ultimately, using a multidisciplinary methodology, this study provides *in vitro* data on the health-promoting properties of FPH, which may serve as a foundation for the development of novel dietary supplements and/or functional foods. We are aware, that an *in vivo* investigation on animal models is needed to achieve the proof of concept for the safety and efficacy of the hydrolysate, but elucidation of the mechanism of action through which FPH exert the multifunctional bioactivity is decisive for designing sustainable and more ethical *in vivo* studies. Based on this consideration, overall, while this study offers valuable preliminary insights into the potential mechanisms of action and bioactive properties of FPH, it may not fully capture the complexities of human metabolism and physiological conditions. To gain a comprehensive understanding of the efficacy and bioavailability of FPH in real-world scenarios, future studies must incorporate *in vivo* models and clinical trials. However, the *in vitro* approach is crucial for screening ingredients with multifunctional behavior and assessing their mechanisms of action prior to conducting more costly experiments involving animals or humans. From ethical and sustainability perspectives, a robust *in vitro* framework serves as an important first step to acquire knowledge that can be further explored in *in vivo* and clinical settings. This approach not only enhances research efficiency but also aligns with ethical practices in scientific studies.

## Data Availability

The raw data supporting the conclusions of this article will be made available by the authors, without undue reservation.
