# Supplementary figures and images for: Effect of ultrasound treatment on quality parameters and health promoting activity of fish protein hydrolysates extracted from side streams of Atlantic mackerel (Scomber scombrus)
Source: Front Nutr. 2024 Sep 4;11:1446485. doi: 10.3389/fnut.2024.1446485 (PMC11408299; doi:10.3389/fnut.2024.1446485)

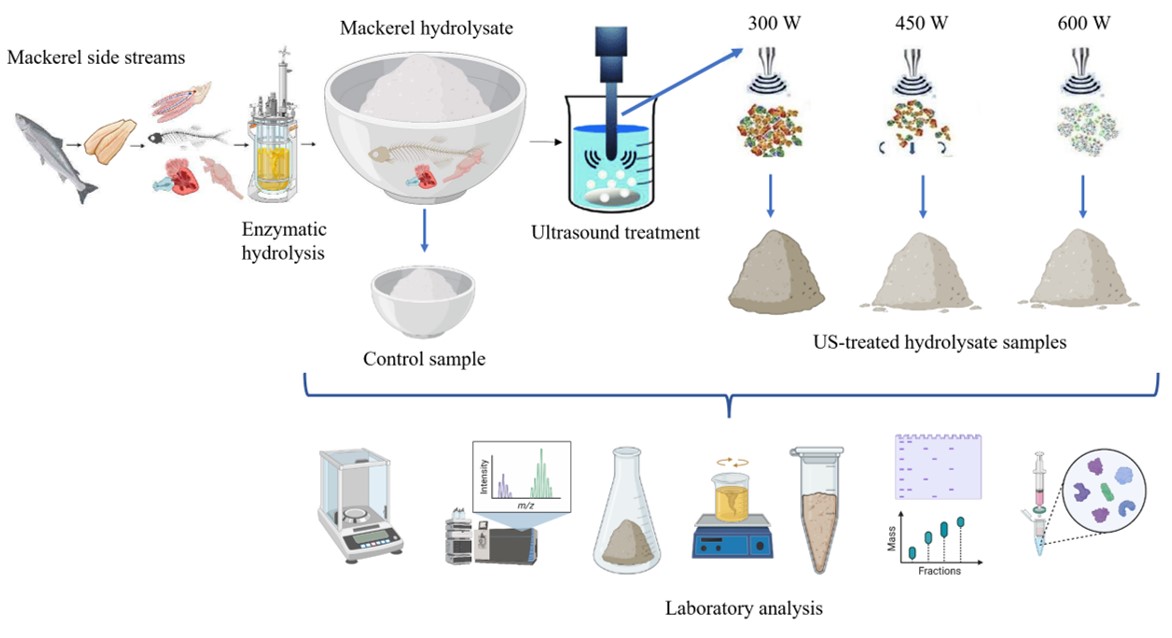

Supplement: Supplementary file 1 [file Image_1.JPEG]
